# Investing in the future of Nursing: the urgent need for sustainable and innovative work environment

**DOI:** 10.1590/1518-8345.0000.4128

**Published:** 2024-11-04

**Authors:** Renata Cristina Gasparino, Olga Maria Pimenta Lopes Ribeiro, José Luís Guedes dos Santos, Andrea Bernardes

**Affiliations:** ^1^ Universidade Estadual de Campinas, Faculdade de Enfermagem, Campinas, SP, Brazil.; ^2^ Escola Superior de Enfermagem do Porto, Porto, Portugal.; ^3^ Universidade Federal de Santa Catarina, Departamento de Enfermagem, Santa Catarina, SC, Brazil.; ^4^ Universidade de São Paulo, Escola de Enfermagem de Ribeirão Preto, PAHO/WHO Collaborating Centre for Nursing Research Development, Ribeirão Preto, SP, Brazil.

**Figure:**
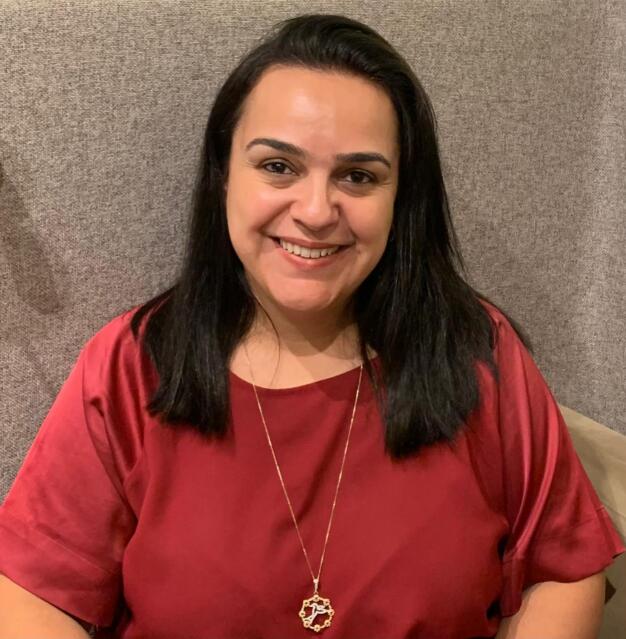

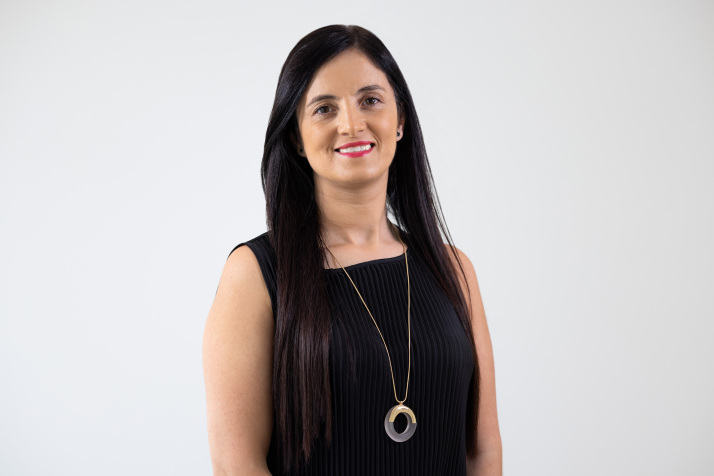

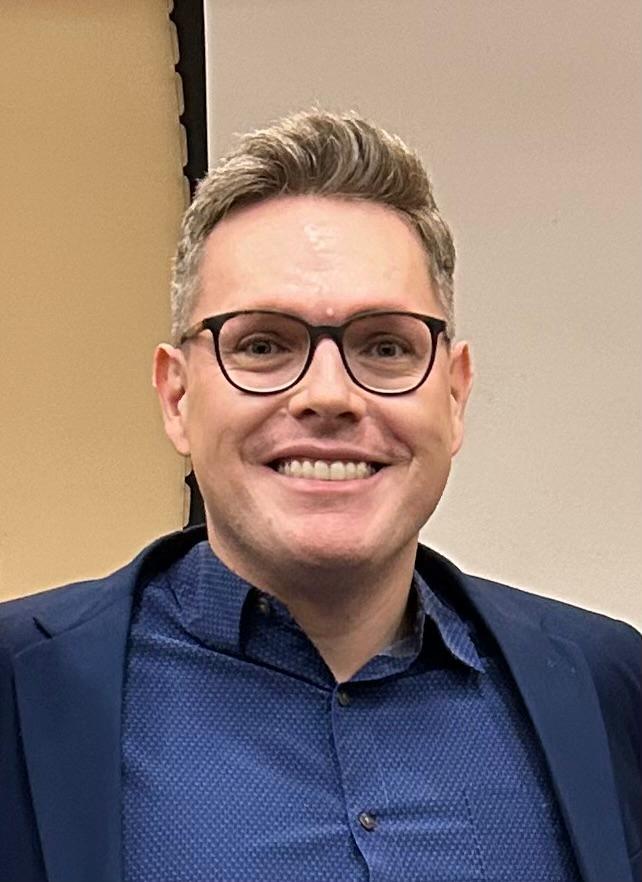

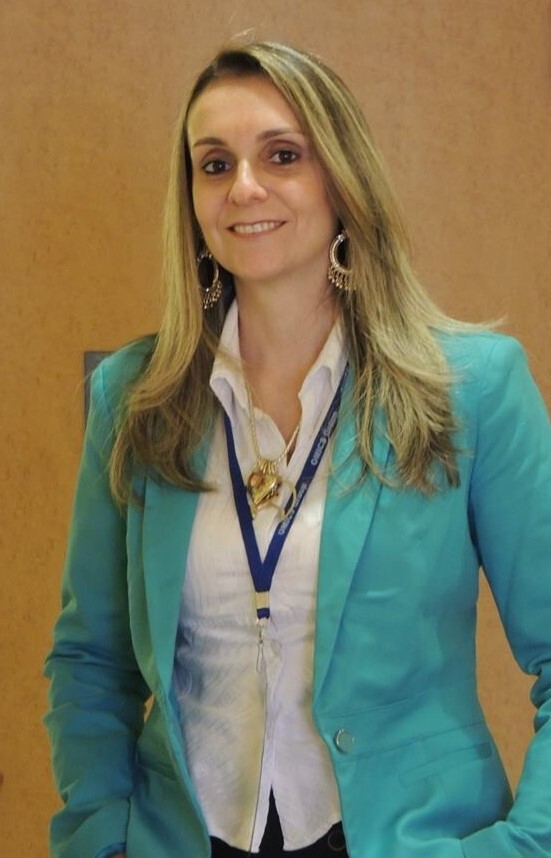

 Different organizations and researchers have recommended that managers take a more cautious look at the work environment. The International Labour Organization recognizes the importance of decent work, understood as one that offers fair wages, safe environments, opportunities for personal development, social integration and protection, freedom of expression and meaningful participation in decision-making. This understanding stands out as an essential element for the effective achievement of globalization and poverty reduction on a global scale ^(^ 1 ^)^ . 

 The World Health Organization (WHO) and the Pan American Health Organization (PAHO) recognize the importance of the relationship between the characteristics of work environments and the most varied outcomes. Therefore, for the decade 2015-2025, they recommend that institutions implement initiatives aimed at healthy and respectful work environments in order to promote the general well-being and quality of life of workers ^(^ 2 ^)^ . 

 The Sustainable Health Agenda for the Americas 2018-2030 emphasizes that managers need to aim to promote healthy, safe and risk-free work environments for workers ^(^ 3 ^)^ , as does the *Política Nacional de Saúde do Trabalhador e da Trabalhadora* , published by the Brazilian *Ministério da Saúde* in 2012. The United Nations, when addressing the Sustainable Development Goals, declares the relevance of the eighth goal – Decent work and economic growth, as it is broadly rooted in the goals of many of the other 16 goals ^(^ 4 ^)^ . 

Regarding the topic, it is important to emphasize that the advent of the COVID-19 pandemic brought a paradox, because, at the same time that the value of nursing professionals became more evident, dissatisfaction, burnout, the intention to leave the job or even the profession became more frequent, in all countries.

Nursing professionals were asked to work in highly complex and uncertain environments, where all individual resources had to be mobilized in order to quickly adapt to the numerous changes. In this sense, as in any recovery/reconstruction process, it is even more essential to act to ensure better working conditions and strengthening of teams, which for at least two years have been subjected to unprecedented professional fatigue, with consequent personal repercussions.

 Investment in the environment in which nursing develops its work processes is fundamental, as there is evidence that characteristics such as leadership skills, foundations aimed at quality of care, nursing participation in decision-making, friendly relationships between teams, adequacy of resources, autonomy, control over the environment, organizational support, communication skills, true collaboration and significant recognition have a positive relationship with different organizational results ^(^ 5 ^)^ . 

These organizational results are described in the literature as improvements in the quality and safety of care received by patients, reduction in mortality rates, higher levels of professional satisfaction, lower levels of burnout, emotional exhaustion and bullying, as well as lower rates of absenteeism and professionals’ intention to leave their jobs.

For transformations in the environment to become a reality, the priorities of researchers and managers must be focused on mapping the presence of these characteristics, with subsequent implementation and evaluation of strategies that redefine the environment.

The central focus of investments is on the financial viability of organizations. Ultimately, by prioritizing the reconfiguration of environments, managers not only strengthen nursing practice but also cultivate an environment that provides comprehensive benefits for everyone involved in the process.

When adding the concerns of strategic international bodies to the research promoted in the area of nursing, it is urgent to highlight that preventive interventions require collaborative and coordinated actions with all productive sectors with the aim of protecting and safeguarding the health and lives of workers, in addition to strengthening public policies and the implementation of new regulations that protect their health.

It is important to underline, however, that the path to (re)constructing work environments is not always synonymous with financial investment. Changes in attitudes and behaviors, and everyone’s involvement in the commitment to build fair, safe and dignified work environments that, in addition to promoting better results for patients/users, ensure harmony between social, personal and organizational well-being, are essential.

Aware of the quality and safety of care provided to people and the optimization of institutional performance, it is crucial that the characteristics of work environments promote the reconciliation between the personal, family, professional and scientific lives of nursing workers, who, given generational diversity, although they share the same work environments, do not necessarily have the same needs.

The complexity of contexts, technological and scientific advances, the growing increase in the population’s demands for care and the constant turnover of nursing teams highlight the need for continuous actions over time, which not only face the challenges imposed by a dynamic and demanding environment, but also recognize the importance of maintaining the quality and effectiveness of nursing services. By ensuring constant commitment, nurses and organizations can make a significant contribution to the continuous improvement of healthcare, strengthening positive outcomes.
