# Supplementary material for: Investing in the future of Nursing: the urgent need for sustainable and innovative work environment
Source: Rev Lat Am Enfermagem. 2024 Nov 4;32:e4128. doi: 10.1590/1518-8345.0000.4128 (PMC11537262; doi:10.1590/1518-8345.0000.4128)
Supplement: Translation

# Translation: Invertir en el futuro de la Enfermería: la necesidad urgente de entornos laborales sostenibles e innovadores

**DOI:** 10.1590/1518-8345.0000.4127

**Published:** 2024-10-17

**Authors:** Renata Cristina Gasparino, Olga Maria Pimenta Lopes Ribeiro, José Luís Guedes dos Santos, Andrea Bernardes

**Affiliations:** ^1^ Universidade Estadual de Campinas, Faculdade de Enfermagem, Campinas, SP, Brasil.; ^2^ Escola Superior de Enfermagem do Porto, Porto, Portugal.; ^3^ Universidade Federal de Santa Catarina, Departamento de Enfermagem, Santa Catarina, SC, Brasil.; ^4^ Universidade de São Paulo, Escola de Enfermagem de Ribeirão Preto, Centro Colaborador de la OPS/OMS para el Desarrollo de la Investigación en Enfermería, Ribeirão Preto, SP, Brasil.

 Diferentes organismos e investigadores han recomendado que los gestores observen con más cautela el entorno laboral. La Organización Internacional del Trabajo reconoce la importancia del empleo decente, entendido como aquel que ofrece salarios justos, entornos seguros, oportunidades para el desarrollo personal, integración y protección social, libertad de expresión y participación significativa en la toma de decisiones. Esta comprensión se destaca como un elemento esencial para el logro efectivo de la globalización y de la reducción de la pobreza a escala global ^(^ 1 ^)^ . 

 La Organización Mundial de la Salud (OMS) y la Organización Panamericana de la Salud (OPS) reconocen la importancia de la relación entre las características de los entornos laborales y los más variados resultados. Por ello, para la década 2015-2025, recomiendan que las instituciones implementen iniciativas encaminadas a ambientes laborales saludables y respetuosos con el fin de promover el bienestar general y la calidad de vida de los trabajadores ^(^ 2 ^)^ . 

 La Agenda de Salud Sostenible para las Américas 2018-2030 enfatiza que los gestores deben apuntar a promover ambientes de trabajo saludables, seguros y libres de riesgos para los trabajadores ^(^ 3 ^)^ , al igual que la *Política Nacional* *de Saúde do Trabalhador e da Trabalhadora* , publicada por el *Ministério da Saúde* de Brasil en 2012. Las Naciones Unidas, al abordar los Objetivos de Desarrollo Sostenible, declaran la relevancia del octavo objetivo – Trabajo decente y crecimiento económico, ya que está ampliamente arraigado en las metas de muchos de los otros 16 objetivos ^(^ 4 ^)^ . 

 Respecto al tema, es importante resaltar que la llegada de la pandemia de COVID-19 trajo una paradoja, pues, al mismo tiempo que se hacía más evidente el valor de los profesionales de enfermería, la insatisfacción, el *burnout* , la intención de dejar el trabajo o incluso la profesión se han vuelto más frecuentes en todos los países. 

Se pidió a los profesionales de enfermería que trabajaran en entornos de alta complejidad e incertidumbre, donde era necesario movilizar todos los recursos individuales para adaptarse rápidamente a innumerables cambios. En este sentido, como en cualquier proceso de recuperación/reconstrucción, es aún más fundamental actuar para garantizar mejores condiciones de trabajo y fortalecer los equipos, que durante al menos dos años se vieron sometidos a un desgaste profesional sin precedentes, con las consiguientes repercusiones personales.

 La inversión en el ambiente en el que la enfermería desarrolla sus procesos de trabajo es fundamental, ya que existe evidencia de que características como la capacidad de liderazgo, los fundamentos orientados a la calidad del cuidado, la participación de la enfermería en la toma de decisiones, las relaciones amistosas entre equipos, la adecuación de los recursos, la autonomía, el control sobre el entorno, el apoyo de la organización, las habilidades de comunicación, la colaboración verdadera y el reconocimiento significativo tienen una relación positiva con diferentes resultados organizacionales ^(^ 5 ^)^ . 

 Estos resultados organizacionales se describen en la literatura como una mejora en la calidad y seguridad de la atención recibida por los pacientes, menores tasas de mortalidad, mayores niveles de satisfacción profesional, menores niveles de *burnout* , agotamiento emocional y *bullying* , así como menores tasas de ausentismo e intención de los profesionales de dejar sus trabajos. 

Para que las transformaciones en el entorno se hagan realidad, las prioridades de investigadores y gestores deben centrarse en mapear la presencia de estas características, con la posterior implementación y evaluación de estrategias que redefinan el ambiente.

El foco central de las inversiones está en la viabilidad financiera de las organizaciones. En última instancia, al priorizar la reconfiguración de los entornos, los gestores no sólo fortalecen la práctica de enfermería, sino que también cultivan un ambiente que proporciona beneficios integrales para todos los involucrados en el proceso.

Al sumar las inquietudes de organismos internacionales estratégicos a las investigaciones promovidas en el área de enfermería, resulta urgente resaltar que las intervenciones preventivas requieren acciones colaborativas y coordinadas con todos los sectores productivos con el fin de proteger y salvaguardar la salud y la vida de los trabajadores, además de fortalecer las políticas públicas y la implementación de nuevas regulaciones que protejan su salud.

Es importante destacar, sin embargo, que el camino hacia la (re)construcción de entornos laborales no siempre es sinónimo de inversión financiera. Son esenciales los cambios de actitudes y comportamientos, y la implicación de todos en el compromiso de construir entornos de trabajo justos, seguros y dignos que, además de promover mejores resultados para los pacientes/usuarios, garanticen la armonía entre el bienestar social, personal y organizacional.

Conscientes de la calidad y seguridad de los cuidados brindados a las personas y de la optimización del desempeño institucional, es fundamental que las características de los ambientes laborales promuevan la conciliación entre la vida personal, familiar, profesional y científica de los trabajadores de enfermería, quienes, ante la diversidad generacional, aunque compartan los mismos entornos de trabajo, no necesariamente tienen las mismas necesidades.

La complejidad de los contextos, los avances tecnológicos y científicos, el creciente aumento de las demandas de cuidados de la población y la constante rotación de los equipos de enfermería resaltan la necesidad de acciones continuas en el tiempo, que no sólo enfrentan los desafíos que impone un entorno dinámico y exigente, sino que también reconocen la importancia de mantener la calidad y eficacia de los servicios de enfermería. Al garantizar un compromiso constante, las enfermeras y las organizaciones pueden contribuir significativamente a la mejora continua de los cuidados de salud, fortaleciendo los resultados positivos.

# Translation: Investindo no futuro da Enfermagem: a necessidade urgente de ambientes de trabalho sustentáveis e inovadores

**DOI:** 10.1590/1518-8345.0000.4129

**Published:** 2024-10-17

**Authors:** Renata Cristina Gasparino, Olga Maria Pimenta Lopes Ribeiro, José Luís Guedes dos Santos, Andrea Bernardes

**Affiliations:** ^1^ Universidade Estadual de Campinas, Faculdade de Enfermagem, Campinas, SP, Brasil.; ^2^ Escola Superior de Enfermagem do Porto, Porto, Portugal.; ^3^ Universidade Federal de Santa Catarina, Departamento de Enfermagem, Santa Catarina, SC, Brasil.; ^4^ Universidade de São Paulo, Escola de Enfermagem de Ribeirão Preto, Centro Colaborador da OPAS/OMS para o Desenvolvimento da Pesquisa em Enfermagem, Ribeirão Preto, SP, Brasil.

 Diferentes órgãos e pesquisadores têm recomendado um olhar mais cauteloso dos gestores para o ambiente de trabalho. A Organização Internacional do Trabalho reconhece a importância de um emprego digno, compreendido como aquele que oferece salários justos, ambientes seguros, oportunidades para o desenvolvimento pessoal, integração e proteção social, liberdade de expressão e participação significativa na tomada de decisões. Este entendimento se destaca como elemento essencial para o alcance efetivo da globalização e da redução da pobreza em escala mundial ^(^ 1 ^)^ . 

 A Organização Mundial da Saúde (OMS) e a Organização Pan-Americana da Saúde (OPAS) reconhecem a importância da relação entre as características dos ambientes de trabalho e os mais variados desfechos. Por isso, para o decênio 2015-2025, recomendam que as instituições implementem iniciativas voltadas para ambientes de trabalho saudáveis e respeitosos a fim de fomentar o bem-estar geral e a qualidade de vida dos trabalhadores ^(^ 2 ^)^ . 

 A Agenda de Saúde Sustentável para as Américas 2018-2030 enfatiza que os gestores precisam ter como meta a promoção de ambientes de trabalho saudáveis, seguros e sem riscos para os trabalhadores ^(^ 3 ^)^ , assim como a Política Nacional de Saúde do Trabalhador e da Trabalhadora, publicada pelo Ministério da Saúde do Brasil em 2012. As Nações Unidas, ao abordarem os Objetivos do Desenvolvimento Sustentável, declaram a relevância do oitavo objetivo – Trabalho decente e crescimento econômico, em decorrência dele estar amplamente enraizado nas metas de muitos dos outros 16 objetivos ^(^ 4 ^)^ . 

 No que diz respeito ao tema, importante reforçar que o advento da pandemia COVID-19 trouxe um paradoxo, pois, ao mesmo tempo em que o valor dos profissionais de enfermagem se tornou mais evidente, a insatisfação, o *burnout* , a intenção de deixar o emprego ou até mesmo a profissão tornaram-se mais frequentes, em todos os países. 

Os profissionais de enfermagem foram solicitados a atuar em ambientes de elevada complexidade e incerteza, onde todos os recursos individuais tiveram que ser mobilizados, no sentido de uma adaptação rápida às inúmeras mudanças. Nessa sequência, como em qualquer processo de recuperação/reconstrução, é ainda mais fundamental atuar para assegurar melhores condições de trabalho e fortalecimentos das equipes, que durante ao menos dois anos estiveram submetidas a um desgaste profissional inédito, com consequentes repercussões pessoais.

 O investimento no ambiente no qual a enfermagem desenvolve seus processos de trabalho é fundamental, pois há evidências de que características como habilidade da liderança, fundamentos dirigidos para a qualidade do cuidado, participação da enfermagem na tomada de decisão, relações amistosas entre as equipes, adequação de recursos, autonomia, controle sobre o ambiente, suporte da organização, habilidades de comunicação, colaboração verdadeira e reconhecimento significativo têm relação positiva com diferentes resultados organizacionais ^(^ 5 ^)^ . 

 Esses resultados organizacionais estão descritos na literatura como melhoria da qualidade e da segurança da assistência recebida pelos pacientes, redução das taxas de mortalidade, maiores níveis de satisfação profissional, menores níveis de *burnout,* exaustão emocional e *bullying* , além de menores taxas de absenteísmo e intenção dos profissionais em se desligar do emprego. 

Para que transformações no ambiente possam se tornar uma realidade, as prioridades de pesquisadores e gestores devem estar voltadas para o mapeamento da presença dessas características, com posterior implementação e avaliação de estratégias que redefinam o ambiente.

O foco central dos investimentos incide na viabilidade financeira das organizações. Em última análise, ao priorizar a reconfiguração dos ambientes, os gestores não apenas fortalecem a prática da enfermagem, mas também cultivam um ambiente que propicia benefícios abrangentes para todos os envolvidos no processo.

Ao somar as preocupações dos órgãos internacionais estratégicos às pesquisas promovidas na área da enfermagem, é premente destacar que as intervenções preventivas requerem ações colaborativas e coordenadas com todos os setores produtivos com o intuito de proteger e resguardar a saúde e a vida dos trabalhadores, além de fortalecer as políticas públicas e a implementação de novas regulamentações que protejam sua saúde.

Importa ressaltar, contudo, que o caminho para a (re)construção dos ambientes de trabalho nem sempre é sinônimo de investimento financeiro. Mudanças de atitudes e comportamentos e envolvimento de todos frente ao compromisso de construir ambientes de trabalho justos, seguros e dignos, que além de promoverem melhores resultados para os pacientes/usuários, garantam harmonia entre o bem-estar social, pessoal e organizacional, são essenciais.

A par da qualidade e segurança dos cuidados prestados às pessoas e da otimização do desempenho institucional, é fundamental que as características dos ambientes de trabalho promovam a conciliação entre a vida pessoal, familiar, profissional e científica dos trabalhadores de enfermagem, que face à diversidade geracional, embora partilhem os mesmos ambientes de trabalho, não têm necessariamente as mesmas necessidades.

A complexidade dos contextos, o avanço tecnológico e científico, o crescente aumento das demandas de cuidados da população e a constante rotatividade das equipes de enfermagem evidenciam a necessidade de ações contínuas ao longo do tempo, que não apenas enfrentem os desafios impostos por um ambiente dinâmico e exigente, mas também reconheçam a importância da manutenção da qualidade e eficácia dos serviços de enfermagem. Ao assegurar um comprometimento constante, os enfermeiros e as organizações podem contribuir significativamente para a melhoria contínua dos cuidados de saúde, fortalecendo os resultados positivos.
